# Supplementary material for: Seeking the Amygdala: Novel Use of Diffusion Tensor Imaging to Delineate the Basolateral Amygdala
Source: Biomedicines. 2023 Feb 13;11(2):535. doi: 10.3390/biomedicines11020535 (PMC9953214; doi:10.3390/biomedicines11020535)

Supplemental Figures

Supplemental Figure S1. MR Contrast-to-noise ratios (CNR) reveal optimal diffusion tensor directionality for identification of the left and right basolateral complex of the amygdala. Raw diffusion tensor images (30 directions) at high-field (11.7 T) illustrate the changing image contrast within the brain and the BLA for providing anatomical specificity in coronal sections from acquired DTI direction. Specific single-direction gradients expose signal loss present both unilaterally and bilaterally within the BLA (Left BLA= yellow, Right BLA = green, combined left and right amygdala = red).

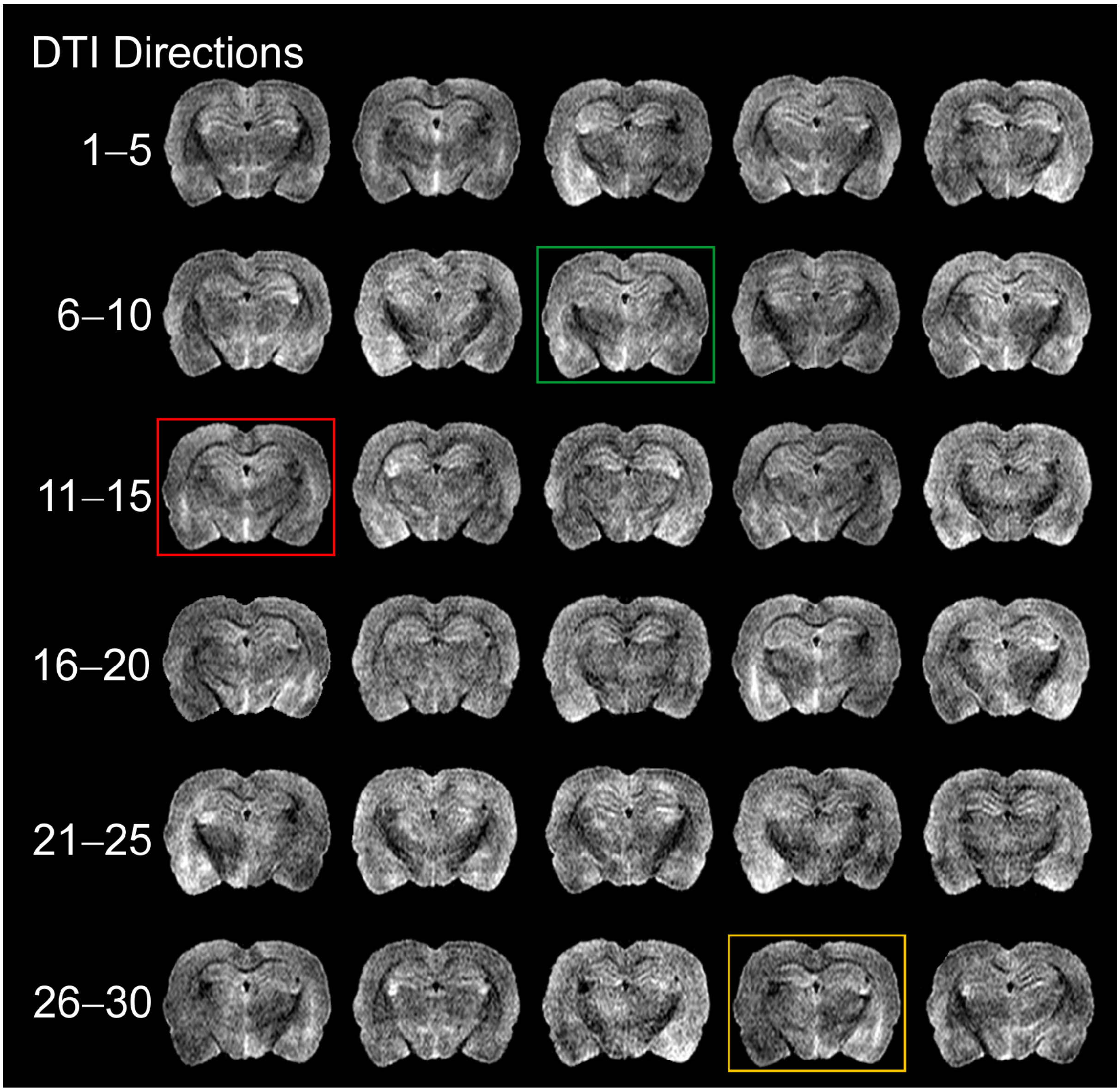

Supplemental Figure S2. High CNR results in better BLA delineation than low CNR DTI directions. Our BLA analyses were based not only on visual inspection, but also from quantitative analysis of CNR within the region delineated by the BLA. It is apparent that low CNR in diffusion directions have no clear BLA boundaries whereas those diffusion directions with high CNR measures have BLA regions that are more discernable. Thus, there are selected DTI directions that are preferential for visualization of the BLA and that are amenable for ROI analysis.

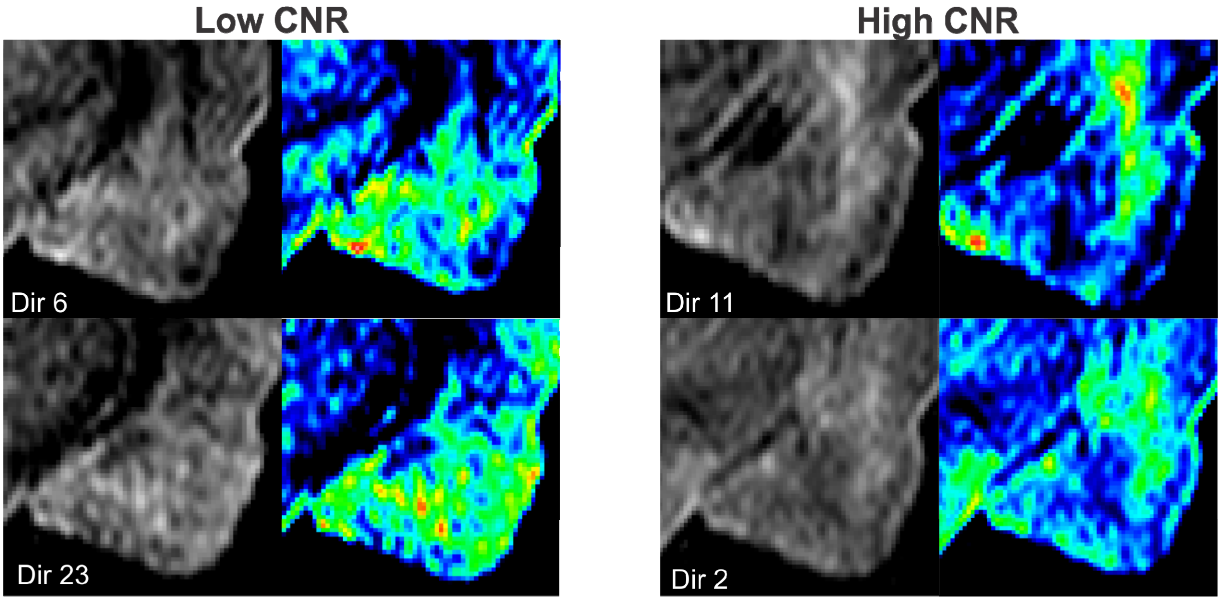

Supplemental Figure S3. DTI slice shift method results in superior accuracy of BLA volumes. A step-wise methodological clarification of the slice shifting DTI method.

a) Acquire acquisition 1 (3 slices, 600um thick, orange) through the BLA.
b) Acquire acquisition 2 (identical to acquisition 1, but shift the slice packet by 200um)
c) Acquire acquisition 3 (identical to acquisition 1, but shift the slice packet by 200um from the location in acquisition 2) (upper panel of the figure illustrates this process).
d) A total of 9 slices (3 slices, 3 acquisitions) are now acquired where the first slice of Acq1 is x=0 um through the BLA, the first slice of Acq2 is now x=200 um into the BLA, and the first slice of Acq3 is x=400 um into the BLA. Continuing, slice 2 of Acq1 is x=600 um, slice 2 of Acq2 is now x=800um and slice two of Acq3 is now x=1000 um into the BLA. Slice 3 of Acq1 repeats at x=1200 um, Acq2 is x=1400 um and Acq 3 is x=1600 um. This approach gives full coverage to the BLA in 200um steps allowing for dramatically increased volumetric assessment of the BLA (relative to the initial 600um data collection).
e) Step 2 (lower panel is to concatenate each of the slice 1's from each of the Acq1,2,3 followed by slice 2 from Acq1,2,3 and then followed by slice 3 from Acq1,2,3 as illustrated using an in house MATLAB routine.
f) The resultant file now has 9 slices spanning 1800 um (effective 1800um, 9 slices at 200um steps) of the BLA for volumetric analysis.
g) The final step is to delineate the BLA boundaries and extract the area of region and sum for volumetric values (see Figure 6 for the results from this method).

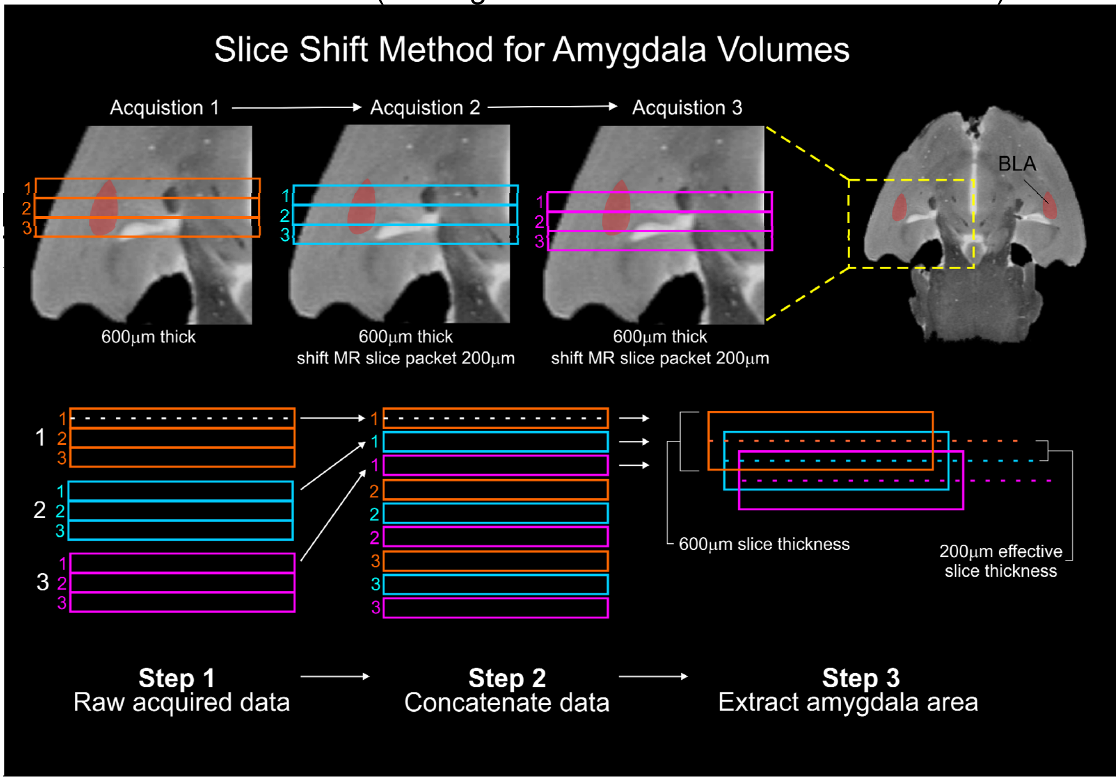

Supplemental Figure S4. Inter-rater measures of BLA volumes. (a) Inter-rater BLA area measures from each MRI slice that encompassed the BLA. Rater A was individual who did the ground truth volume measures, Rater B had limited MRI and no BLA knowledge, whilst Rater C had extensive MRI but limited BLA experience. While Raters A and C had similar area measures, Rater B over-estimated the BLA boundaries resulting in a 50.6% increase in BLA volumes relative to Rater A. These data are prior to a training session for Rater B. After training Rater B reported BLA volumes of 14.6% larger than Rater A (see Figure 6f). **(b)** Regions of interest drawn by the raters on this slice: Raters: A (red), B (yellow), C (blue).

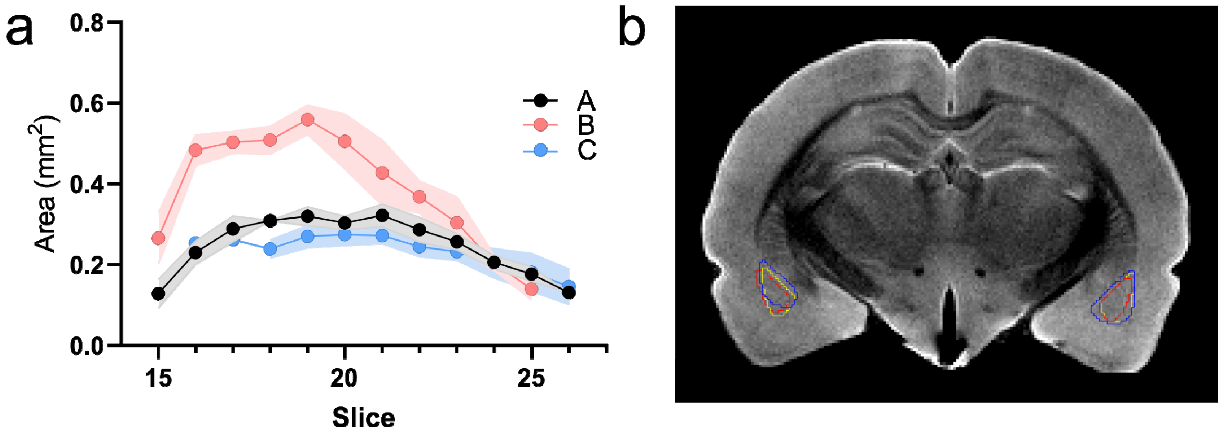

Supplement: Supplementary file 1 [file biomedicines-11-00535-s001.zip › Supplemental Figures File 012323AJao.pdf]
